# Novel removable endoscopic clip: Usefulness in failure of traction method during endoscopic submucosal dissection

**DOI:** 10.1055/a-2155-5377

**Published:** 2023-09-15

**Authors:** Nobukazu Agatsuma, Takahiro Utsumi, Hirokazu Higuchi, Takahiro Inoue, Yukari Tanaka, Yuki Nakanishi, Hiroshi Seno

**Affiliations:** 1 Department of Gastroenterology and Hepatology, Graduate School of Medicine, Kyoto University, Kyoto, Japan; 2 Department of Medical Supply, Kyoto University Hospital, Kyoto, Japan

Endoscopic clips, which are widely used for hemostasis and closure 1 2 , have recently been applied to other indications such as traction-assisted endoscopic submucosal dissection (ESD) 3 . Repositionable clips have been reported to be a promising option for any indication 4 5 . Despite the adoption of clips that allow repeated opening and closing before clip deployment, endoscopists still encounter situations in which they want to remove the placed clip. Here, we present a novel removable clip (hemoclip, AG-51044-2300-090-16; Hangzhou AGS MedTech Co., Ltd., Hangzhou, China) that is repositionable and rotatable. The clip was detached by squeezing the thinning point at the end of the clip stem using a polypectomy snare ( Fig. 1 ).

**Fig. 1:**
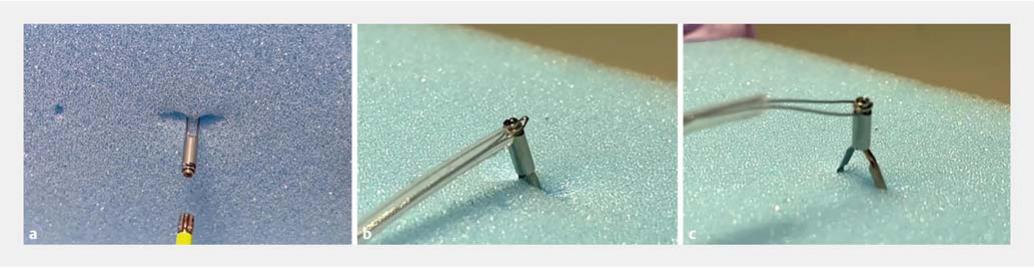
How to remove the hemoclip, a novel removable clip. **a** The hemoclip deployed on the mucosa. **b** Squeezing the thinning point at the end of the clip stem using a polypectomy snare. **c** The clip being immediately detached from the mucosa.

This video shows the usefulness of novel removable endoscopic clips in failure of the traction method during gastric ESD in in vivo porcine models ( Video 1 ). The new clip, using a clip-with-line method, accidentally grasped both the edge of the lesion and the muscle layer, making it difficult to continue ESD safely. However, the mistakenly placed clip was able to be easily removed using a polypectomy snare (AG-5078–241023; Hangzhou AGS MedTech Co., Ltd) ( Fig. 2 ). Then, a clip with a ring-loaded spring (S-O clip, TC1H05; Zeon Medical Co., Ltd., Tokyo, Japan) was used for the traction method during ESD. The removable clip captured the loop part of the S-O clip and anchored it to the gastric wall. However, the misplaced clip did not provide a good field of vision or adequate tension in the submucosal dissection plane. The clip was removed using a snare, and a new clip was anchored to the proper position ( Fig. 3 ). It is possible to perform the procedure again using a removable clip, even when closing the clip results in a risky or ineffective situation.

**Fig. 2:**
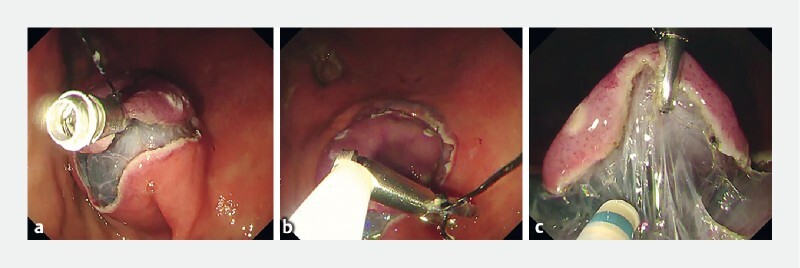
Removal of the hemoclip, which mistakenly grasped the muscle layer in the clip-with-line method. **a** The hemoclip accidentally grasped both the edge of the lesion and the muscle layer. **b** The clip being removed from the muscle layer using a polypectomy snare. **c** The new clip being deployed without grasping the muscle layer, resulting in a good field of vision and adequate tension for endoscopic submucosal dissection.

**Fig. 3:**
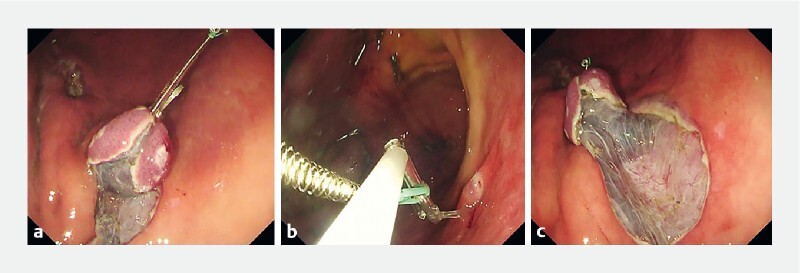
The removal of the hemoclip allows the S-O clip traction direction to be changed many times. **a** Although the hemoclip captures the loop of the S-O clip and anchors it to the gastric wall, the traction tension is inadequate for submucosal dissection. **b** The hemoclip is easily removed using a polypectomy snare. **c** The new clip is anchored to another site on the gastric wall, resulting in a good field of vision and adequate tension for endoscopic submucosal dissection.

**Video 1** Demonstration of the usefulness of novel removable endoscopic clips in failure of the traction method during endoscopic submucosal dissection in a porcine model.


Endoscopy_UCTN_Code_TTT_1AQ_2AD

## Notes

Endoscopy E-Videos

https://eref.thieme.de/e-videos
*E-Videos* is an open access online section of the journal *Endoscopy* , reporting on interesting cases and new techniques in gastroenterological endoscopy. All papers include a high-quality video and are published with a Creative Commons CC-BY license. Endoscopy E-Videos qualify for HINARI discounts and waivers and eligibility is automatically checked during the submission process. We grant 100% waivers to articles whose corresponding authors are based in Group A countries and 50% waivers to those who are based in Group B countries as classified by Research4Life (see: https://www.research4life.org/access/eligibility/ ). This section has its own submission website at https://mc.manuscriptcentral.com/e-videos
